# Factors influencing oocyte yield and embryo quality in donor IVF cycles: a retrospective cohort study

**DOI:** 10.3389/fendo.2025.1649523

**Published:** 2025-10-31

**Authors:** Pin-Yao Lin, Chun-I Lee, Hsiu-Hui Chen, Chun-Chia Huang, Ming-Jer Chen, Tzu-Ning Yu, Tsung-Hsien Lee, Maw-Sheng Lee

**Affiliations:** ^1^ Division of Infertility, Lee Women’s Hospital, Taichung, Taiwan; ^2^ Department of Post-Baccalaureate Medicine, National Chung Hsing University, Taichung, Taiwan; ^3^ Institute of Medicine, Chung Shan Medical University, Taichung, Taiwan; ^4^ Department of Obstetrics and Gynecology, Chung Shan Medical University Hospital, Taichung, Taiwan; ^5^ Department of Obstetrics and Gynecology and Women’s Health, Taichung Veterans General Hospital, Taichung, Taiwan; ^6^ School of Medicine, National Yang-Ming Chiao Tung University, Taipei, Taiwan

**Keywords:** oocyte donation, anti-Müllerian hormone, progestin-primed ovarian stimulation, body mass index, dual trigger, donor *in vitro* fertilization (IVF), propensity score matching

## Abstract

**Background:**

Achieving an optimal balance between oocyte yield and embryo quality is central to donor IVF. Although anti-Müllerian hormone (AMH) predicts oocyte quantity, donor characteristics and stimulation parameters may influence embryo developmental competence. We aimed to identify clinical and protocol-related factors associated with oocyte yield and embryo quality in a large donor cohort.

**Methods:**

We retrospectively analyzed 584 donor IVF cycles at a single center (Jan 1, 2018–Dec 31, 2023). Donor variables included age, AMH, BMI (body mass index), and baseline hormones. Stimulation used gonadotropin-releasing hormone (GnRH) antagonist (27.1%) or progestin-primed ovarian stimulation (PPOS)(72.9%); recombinant human luteinizing hormone (LH) was used in 86% of cycles; final maturation was GnRH agonist (GnRHa) (59.4%) or dual trigger (40.6%). Outcomes were oocyte yield and embryo quality metrics (maturation, two-pronuclear (2PN) fertilization, Day 3 good-quality embryo rate, blastocyst formation rate, and top-quality blastocyst rate). Multivariable linear regression and propensity score matching (PSM) compared protocols and LH supplementation.

**Results:**

Donors were 25.6 ± 3.7 years with AMH 6.1 ± 2.9 ng/mL and BMI 21.6 ± 2.8 kg/m². Per cycle: 27.1 ± 11.1 oocytes, 20.8 ± 8.3 MII (maturation 78.2 ± 13.4%), 2PN fertilization 73 ± 18%, Day 3 good-quality embryos 10.6 ± 6.0 (69.5 ± 23.5%), blastocysts 9.5 ± 5.5 (56.7 ± 22.5%), and top-quality blastocysts 6.1 ± 4.2 (36.5 ± 20.1%). First transfers (n=491) yielded 55.4% clinical pregnancy and 44.4% live birth; miscarriage 12.2%. AMH independently predicted oocyte number; higher BMI was associated with lower fertilization. PPOS produced lower Day 3 good-quality embryo (67.9% vs 72.8%) and top-quality blastocyst rates (59.7% vs 74.1%) versus antagonist in PSM, with similar blastocyst formation rate (64.0% vs 60.9%). LH supplementation modestly increased fertilization (74.4% vs 69.5%) without downstream differences. Dual trigger was associated with reduced blastocyst formation rate but a higher proportion of top-quality blastocysts.

**Conclusions:**

In donor IVF, AMH is the principal predictor of oocyte yield, whereas BMI, stimulation protocol, and trigger method influence embryo quality. Despite protocol-dependent morphology differences, pregnancy and live birth rates remained high. Findings support individualized stimulation strategies that consider donor profile and protocol effects to optimize efficiency and outcomes.

## 1 Background

Oocyte donor cycles provide a well-controlled model to examine factors influencing ovarian response and oocyte quality, as donors are typically young, healthy, and undergo controlled ovarian stimulation under standardized conditions. While maximizing oocyte yield is essential to meet recipient demand, the quality of retrieved oocytes is equally critical to ensure optimal fertilization, embryo development, and pregnancy outcomes. Although ovarian reserve markers such as anti-Müllerian hormone (AMH) and antral follicle count are well-established predictors of oocyte quantity (1, 2), the extent to which donor characteristics and specific stimulation protocols affect oocyte developmental competence remains less well understood.

Young donors typically yield a high number of oocytes; however, an excessive response may be associated with diminishing per-oocyte viability. Prior studies suggest an optimal range of 12–18 oocytes for maximizing implantation, beyond which embryo quality may plateau or decline (3). Donors often exceed this threshold, raising concerns about whether high responders experience compromised oocyte maturation or embryo potential (4). Higher BMI, even within a non-obese range, has been linked to reduced fertilization and blastocyst development in autologous IVF (5, 6). Whether these findings apply to donor IVF cycles remains unclear.

Beyond donor characteristics, stimulation protocols and cycle management strategies may also impact oocyte and embryo quality (7). The progestin-primed ovarian stimulation (PPOS) protocol is widely used in donor cycles for its scheduling advantages, but its effect on embryo competence is debated. Some studies report pregnancy rates comparable to those achieved with GnRH antagonist protocols (8), while others suggest reduced blastocyst quality in certain populations (9). Similarly, the routine use of r-hLH supplementation in young oocyte donors—who typically have intact hypothalamic-pituitary function—has not consistently demonstrated clinical benefit. Nonetheless, its potential impact on fertilization and embryo development may still warrant investigation, as some studies have reported improved embryo quality and live birth rates with LH supplementation even in GnRH antagonist protocols (10–12). The optimal approach for final oocyte maturation triggering in donors, whether using a GnRH agonist alone or a dual trigger (GnRH agonist plus low-dose hCG), also remains unclear. While dual triggers have demonstrated benefits in specific subgroups, such as poor responders or those with previous suboptimal outcomes (13), evidence in young donor populations is limited.

In summary, while many predictors of oocyte yield are known, their relationship to oocyte and embryo quality in the donor population is not fully elucidated. Subtle differences in donor profile and cycle management may influence outcomes, warranting a comprehensive analysis. In this retrospective cohort study, we aimed to identify which donor characteristics and stimulation protocol factors are associated with oocyte yield and embryo developmental competence in donor IVF cycles.

## 2 Materials and methods

### 2.1 Study design and study population

**Figure 1:**
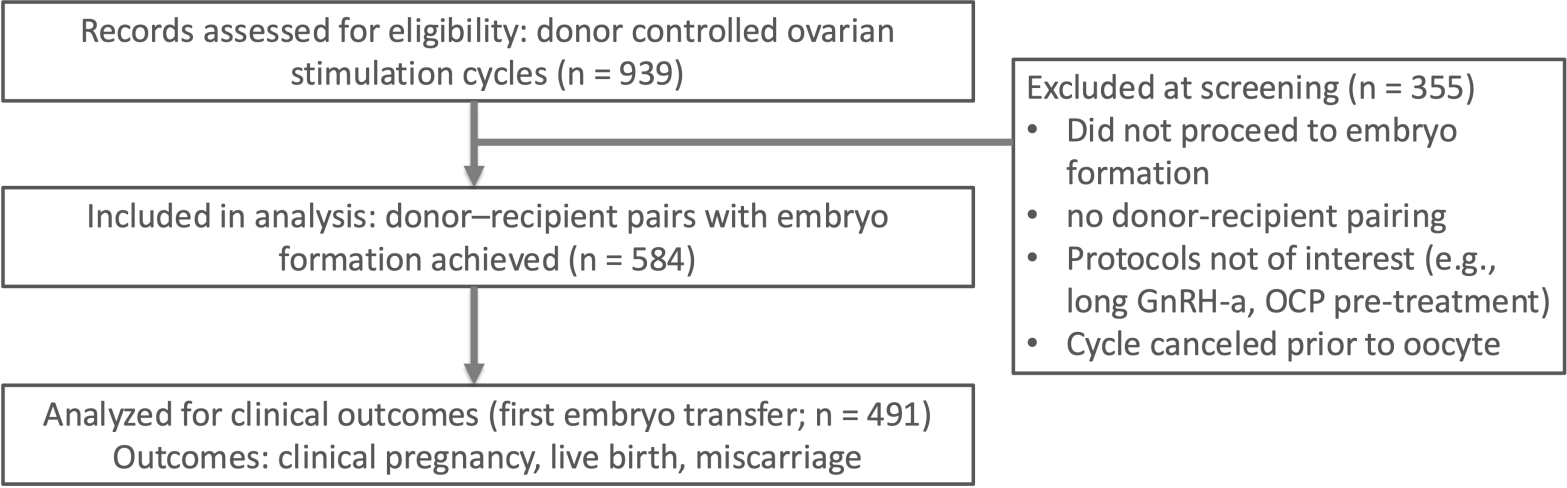
Of 939 donor controlled ovarian stimulation cycles screened, 584 donor–recipient pairs with embryo formation were included in the analysis. A subset of 491 first embryo transfers contributed to clinical outcomes (clinical pregnancy, live birth, miscarriage). Screening exclusions (n = 355) were due to no embryo formation, no donor–recipient pairing, non-study protocols (e.g., long GnRH agonist protocol or OCP pre-treatment), or cycle cancellation before oocyte retrieval. OCP, oral contraceptive pill.

This retrospective study reviewed the records of 939 oocyte donors who underwent ovarian stimulation for egg banking at an IVF clinic in Taiwan between January 1, 2018, and December 31, 2023. (**Figure 1**) Of 939 donor stimulation cycles screened, 584 donor–recipient pairs proceeded to embryo creation and were included in the final analysis; 355 were excluded at screening for procedural reasons (no embryo creation/no pairing; protocol not of interest; cancellation before retrieval) prior to outcome ascertainment.

Only cycles utilizing either a GnRH antagonist or a progestin-primed ovarian stimulation (PPOS) protocol were included. Exclusion criteria comprised the use of long GnRH agonist protocols, pre-treatment regimens such as oral contraceptive scheduling, or cancellation of the cycle prior to oocyte retrieval. In addition to donor records, the clinical outcomes of oocyte recipients whose embryos were derived from these donated oocytes were assessed.

Eligible donors were between 20 and 35 years of age, had regular menstrual cycles (25–35 days), and a BMI between 18 and 25 kg/m². All donors had a normal karyotype, adequate ovarian reserve (AMH > 4 ng/mL), no hereditary disorders, and no history of hormonal treatment in the three months preceding donation. According to Taiwan’s Assisted Reproduction Act, oocytes from a single donor may be assigned to only one recipient, thereby preventing multiple allocations from the same donation. This regulation aims to uphold ethical standards, minimize associated risks, and ensure traceability and accountability in the donation process. This study was approved by the Institutional Review Board of Chung Shan Medical University Hospital (IRB: CS2-20201). Due to its retrospective nature, the requirement for written informed consent was waived.

### 2.2 Controlled ovarian hyperstimulation procedures

Controlled ovarian stimulation was conducted using either a GnRH antagonist or a PPOS protocol. In both protocols, stimulation began on day 3 of the menstrual cycle with 225–300 IU/day of recombinant FSH, either alone or in combination with recombinant LH (r-hFSH + r-hLH; Pergoveris, Merck-Serono, Germany), for five days. Thereafter, 225 IU/day of r-hFSH alone (Gonal-F, Merck-Serono, Germany) was continued until the day of final oocyte maturation triggering.

In the GnRH antagonist group, 0.25 mg of cetrorelix acetate (Cetrotide, Merck-Serono, Germany) was administered daily starting on stimulation day 6 for four consecutive days to suppress premature LH surges. In the PPOS group, oral progestin (medroxyprogesterone acetate, 5 mg twice daily; Provera) was initiated on cycle day 5 and continued until the trigger day.

In both protocols, ovarian response was monitored through serial transvaginal ultrasonography and serum hormone measurements. Final oocyte maturation was triggered when at least two leading follicles reached a mean diameter of ≥17 mm. Triggering was achieved via subcutaneous injection of 0.2 mg triptorelin (GnRH agonist; Decapeptyl, Ipsen, France), either alone or combined with 1500–3000 IU of recombinant human chorionic gonadotropin (hCG) (Ovidrel, Merck, Germany), depending on ovarian response and the risk of ovarian hyperstimulation syndrome (OHSS). Ovarian hyperstimulation syndrome (OHSS) was defined and graded according to the 2024 American Society for Reproductive Medicine (ASRM) Practice Committee guideline on prevention and management of moderate–severe OHSS. For incidence reporting, we present moderate–severe OHSS consistent with the ASRM definitions (14).

### 2.3 *In vitro* fertilization, embryo culture, TE biopsy, and frozen embryo transfer

Oocyte retrieval, fertilization, embryo culture, trophectoderm (TE) biopsy, and embryo vitrification/warming were performed according to established protocols (15). Retrieved oocytes were denuded and classified as metaphase II (MII), metaphase I (MI), or germinal vesicle (GV) stage. Mature oocytes (MII) were vitrified using the Cryotec system (Cryotec, Japan) according to the manufacturer’s protocol. Vitrification and warming followed a standard equilibration and ultra-rapid cooling process as described by Gandhi et al (16). All warmed oocytes underwent intracytoplasmic sperm injection (ICSI). Normally fertilized zygotes were defined by the presence of two pronuclei (2PN). Day 3 good-quality embryos were defined as those with ≥8 cells, <20% fragmentation, and symmetrical blastomeres. Blastocyst grading was performed on day 5 or 6 using the Gardner blastocyst morphological scoring system (17). A blastocyst was considered top-quality if it reached expansion grade ≥3 and had an inner cell mass and trophectoderm graded A or B. Top-quality blastocysts were transferred or cryopreserved for frozen embryo transfer. The first embryo transfer per recipient was analyzed to evaluate clinical outcomes. Endometrial preparation was conducted using either a natural cycle or hormone replacement therapy with estradiol and progesterone. An endometrial thickness of ≥7 mm was required prior to embryo transfer. Recipient outcomes are summarized for the first embryo transfer only. Because the first transfer uses the highest-quality embryo available, protocol-level differences upstream are expected to be minimized at this endpoint.

### 2.4 Data collection and outcome measurements

Two categories of outcomes were evaluated: (a) Oocyte yield: the total number of oocytes retrieved per donor cycle, including all maturation stages. (b) Oocyte and embryo quality outcomes, including: maturation rate = MII oocytes/total oocytes retrieved (%), normal fertilization rate = 2PN zygotes/MII oocytes (%), Day 3 good-quality embryo rate = good-quality cleavage-stage embryos/2PN embryos (%), blastocyst formation rate = blastocysts/2PN embryos (%), top-quality blastocyst rate = top-quality blastocysts/total blastocysts (%). In addition, the clinical pregnancy rate per cycle was recorded and defined as the presence of a gestational sac with a fetal heartbeat detected by ultrasound at 6–8 weeks of gestation following the first embryo transfer, whether from fresh or frozen donor-derived embryos.

Covariates included donor age, AMH, BMI, total administered doses of FSH and LH, serum estradiol (E2) and progesterone (P4) levels on the trigger day, stimulation protocol (GnRH antagonist vs PPOS), LH supplementation (yes/no), and trigger method (GnRH agonist alone, hCG alone, or dual trigger).

### 2.5 Statistical analysis

We summarized donor characteristics and cycle outcomes with descriptive statistics. Between-group comparisons used independent-sample t tests or χ² tests, as appropriate. Multivariable linear regression evaluated predictors of (1) oocyte yield and (2) embryo-quality outcomes (oocyte maturation, 2-pronuclear [2PN] fertilization, Day 3 good-quality embryo, blastocyst formation, and top-quality blastocyst rates). Coefficients are unstandardized and reported with 95% confidence intervals (CI); outcomes are percentages, so each β is interpreted as an absolute percentage-point change per unit of the predictor (e.g., per 1 ng/mL AMH, per 1 kg/m² BMI, per 100 IU total gonadotropin; PPOS vs antagonist; dual trigger vs GnRHa). Skewed variables were log-transformed before analysis. For the five prespecified embryo-quality endpoints, multiplicity was controlled using Bonferroni (family-wise α=0.05; P<0.005 significant). All other analyses are exploratory with P<0.05 reported for context.

To reduce confounding in protocol contrasts, we performed propensity score matching (PSM) for PPOS vs antagonist (1:2; 154 vs 308 cycles) and LH+ vs LH− (1:2; 162 vs 81 cycles). Propensity scores were estimated from donor age, AMH and BMI; matching used nearest-neighbor with caliper = 0.2 × standard deviation (SD) of the logit(PS). To preserve common support and avoid overfitting in a finite matched sample, the propensity-score model was intentionally limited to baseline determinants (age, AMH, BMI). Covariate balance was assessed by standardized mean differences (SMD < 0.10). We conducted sensitivity analyses using stabilized inverse probability weighting with the same covariates, which yielded directionally consistent estimates. (Follicle counts were not uniformly available; peak estradiol and total gonadotropin dose were used as proxies for follicular response.)

For tables, we additionally report absolute between-group differences (expressed in percentage points) with 95% CI for the two-sample difference in means, alongside group summaries and P values. Based on the observed variability of percentage outcomes (SD approximately 22%), the matched protocol comparison (154 vs 308) provides approximately 80–85% power (two-sided α=0.05) to detect an absolute difference of approximately 6 percentage points; effects near this magnitude are interpreted cautiously. All analyses were performed with SPSS v25 (IBM) and R software.

## 3 Results

### 3.1 Baseline characteristics

Among the 939 oocyte donor cycles performed for egg banking, the incidence of suboptimal ovarian response (≤9 oocytes retrieved) was low at 6.4% (n = 61). Moderate to severe ovarian hyperstimulation syndrome (OHSS) occurred in 1.5% of cases, while other complications—including intra-abdominal bleeding, ovarian torsion, infection, procedural injury, or severe pain—were reported in 1.0% of cycles. Of these, 584 donor–recipient pairs who proceeded to oocyte warming and embryo creation were included in the final analysis.

**Table 1** summarizes the clinical characteristics and outcomes of the 584 oocyte donor stimulation cycles. Donors had a mean age of 25.6 years, with an average AMH of 6.1 ± 3.0 ng/mL and BMI of 21.6 kg/m². The majority of cycles (73%) utilized a progestin-primed ovarian stimulation (PPOS) protocol, while 27% employed a GnRH antagonist protocol. LH supplementation was used in 86% of cycles. A GnRH agonist trigger was applied in 60% of cases, and a dual trigger (GnRH agonist plus low-dose hCG) in the remaining 40%. On the trigger day, mean peak estradiol and progesterone levels were 6040 pg/mL and 1.4 ng/mL, respectively.

**Table 1:** Baseline donor characteristics and ovarian stimulation outcomes across 584 cycles.

Oocyte donor cycle characteristics	
Variable	Mean ± SD
No. of cycles	584
Donor Age (years)	25.6 ± 3.7
Anti-Müllerian Hormone (ng/mL)	6.1 ± 2.9
Body Mass Index (kg/m²)	21.6 ± 2.8
Day 3 FSH (mIU/mL) E2 (pg/mL) *LH (mIU/mL)*	6.4 ± 2.4 40.7 ± 47.3 6.3 ± 6.2
LH on trigger day (mIU/mL) *E2 (pg/mL)* *P4 (ng/mL)*	2.3 ± 3.1 6037.9 ± 4089 1.42 ± 1.22
Total FSH Dose (IU)	2406.8 ± 374
Total LH Dose (IU)	1294 ± 701
*Protocol (n; %)* *GnRH antagonist* *PPOS*	158 (27.1%) 426 (72.9%)
LH component use (n; %) LH (+) LH (-)	502 (86%) 82 (14%)
Trigger method GnRHa GnRHa + hCG	347 (59.4%) 237 (40.6%)
Total Retrieved Oocytes (n)	27.1 ± 11.1
MII Oocytes(n)	20.8 ± 8.3
Oocyte Maturation rate (%)	78.2 ± 13.4%
Oocyte thawing survival rate (%)	93.3 ± 13.7%
2PN Fertilization (n, %)	15.1 ± 7 (73 ± 18%)
Day 3 good-quality embryo (n, %)	10.6 ± 6.0 (69.5 ± 23.5%)
Total blastocysts (n, %)	9.5 ± 5.5 (56.7 ± 22.5%)
Top-quality blastocysts (n, %)	6.1 ± 4.2 (36.5 ± 20.1%)
Recipient characteristics and outcomes of donated oocytes	
Recipient Age (years)	42.9 ± 5.6
Body Mass Index (kg/m²)	22.7 ± 3.8
Estradiol on ET day, pg/mL	345 ± 57
Progesterone on ET day, ng/mL	34 ± 23
Endometrial thickness, mm	9.1 ± 0.8
1^st^ embryo transfer ET number Clinical Pregnancy Rate (%, n) Live birth Rate (%, n) Miscarriage, (%, n)	N = 491
	1.28 ± 0.3
	55.4% (272/491)
	44.4% (218/491)
	12.2% (60/491)

Data are presented as mean ± standard deviation or percentage as appropriate.

AMH, anti-Müllerian hormone; BMI, body mass index; FSH, follicle-stimulating hormone; LH, luteinizing hormone; E2, estradiol; P4, progesterone; GnRH ant, gonadotropin-releasing hormone antagonist; PPOS, progestin-primed ovarian stimulation; GnRHa, GnRH agonist; hCG, human chorionic gonadotropin; MII, metaphase II oocyte; 2PN, two-pronuclear zygote. ET, embryo transfer.

Each cycle yielded an average of 27.1 oocytes, with 20.8 reaching the MII stage, corresponding to a mean maturation rate of 78%. An average of 15.1 oocytes fertilized normally (2PN), yielding a fertilization rate of 73%. On Day 3, 10.5 good-quality embryos were obtained per cycle. Blastocyst formation averaged 9.5 per cycle, with a blastocyst formation rate of 63% per 2PN zygote. Of these, 6.1 blastocysts were graded as top-quality, accounting for 64% of all blastocysts. These findings indicate a robust ovarian response and embryo yield in this donor cohort. Recipients had a mean age of 42.9 ± 5.6 years and a BMI of 22.7 ± 3.8 kg/m². At the time of embryo transfer, the mean endometrial thickness was 9.1 mm, and serum estradiol and progesterone levels averaged 345 ± 57 pg/mL and 34 ± 23 ng/mL, respectively.

Among 491 first embryo transfer cycles, an average of 1.28 ± 0.3 embryos were transferred per cycle. Clinical pregnancy and live birth rates were 55.4% (272/491) and 44.4% (218/491), respectively. The miscarriage rate was 12.2% (60/491).

### 3.2 Predictors of oocyte yield

**Table 2** presents the results of univariate and multivariable linear regression analyses identifying predictors of total oocyte yield. In both models, anti-Müllerian hormone (AMH) emerged as the strongest independent predictor (95% CI: 0.81–1.38; P < 0.001). Peak estradiol (E2) levels were also positively associated with oocyte count (β = 0.0010 per pg/mL; P < 0.001). A higher total LH dose showed a modest but statistically significant negative association with oocyte yield in the adjusted model (β = –0.0019 per IU; P = 0.032), suggesting that higher LH requirements may reflect reduced ovarian responsiveness.

**Table 2:** Multivariable linear regression analysis identifying independent predictors of oocyte number.

Predictor	Univariate β (95% CI)	P value	Multivariate β (95% CI)	P value
Donor age (per year)	0.01 (−0.24, 0.26)	0.94	−0.09 (−0.30, 0.11)	0.37
Donor AMH (per ng/mL)	1.72 (1.45, 1.99)	<0.001*	1.10 (0.81, 1.38)	<0.001
Donor BMI (per kg/m²)	−0.24 (−0.57, 0.08)	0.14	−0.13 (−0.40, 0.14)	0.34
Total FSH dose (per IU)	−0.0027 (−0.0052, −0.0003)	0.025	0.0004 (−0.0017, 0.0025)	0.72
Total LH dose (per IU)	−0.0021 (−0.0034, −0.0008)	0.001	−0.0019 (−0.0037, −0.0002)	0.032
Peak E2 (per pg/mL)	0.00135 (0.00116, 0.00154)	<0.001	0.0010 (0.0008, 0.0012)	<0.001
Progesterone at trigger (ng/mL)	2.03 (1.30, 2.75)	<0.001	0.14 (−0.52, 0.81)	0.68
Protocol (PPOS vs GnRH antagonist)	−1.30 (−3.35, 0.75)	0.21	0.31 (−1.61, 2.22)	0.75
LH supplementation (yes vs no)	−2.31 (−4.92, 0.29)	0.082	−0.04 (−3.28, 3.20)	0.98
Trigger method (dual vs GnRHa)	−0.73 (−2.58, 1.11)	0.44	1.23 (−0.31, 2.77)	0.12

β = unstandardized coefficient; values indicate the absolute change in total oocyte number per unit of the predictor (or difference vs reference). Predictor units: AMH (ng/mL), BMI (kg/m²), total FSH dose (IU), total LH dose (IU), peak E2 (pg/mL), trigger-day P4 (ng/mL); protocol (PPOS vs GnRH antagonist [reference]); LH supplementation (yes vs no); trigger (dual vs GnRHa [reference]). Skewed variables were log-transformed before analysis. Two-sided P < 0.05 was considered statistically significant for this table. AMH, anti-Müllerian hormone; BMI, body mass index; FSH, follicle-stimulating hormone; LH, luteinizing hormone; E2, estradiol; P4, progesterone; PPOS, progestin-primed ovarian stimulation; GnRH antagonist, gonadotropin-releasing hormone antagonist; GnRHa, GnRH agonist; CI, confidence interval. *P<0.05.Footnote under Table 2 (final): P<0.05 indicates statistical significance.

In contrast, donor age, BMI, total FSH dose, trigger-day progesterone, stimulation protocol, LH supplementation, and trigger method were not significantly associated with oocyte yield in the multivariable model (P > 0.05 for all). Several variables—such as total gonadotropin doses and progesterone—reached significance in univariate analysis but lost significance after adjustment, indicating potential confounding. These findings underscore AMH and estradiol as key predictors of oocyte yield, while elevated LH dose may indicate decreased ovarian efficiency.

### 3.3 Predictors of oocyte quality outcomes

**Table 3** presents multivariable regression results for five quality outcomes: oocyte maturation rate, 2PN fertilization rate, Day 3 good-quality embryo rate, blastocyst formation rate, and top-quality blastocyst rate.

**Table 3:** Multivariable linear regression analysis identifying predictors of oocyte and embryo quality outcomes.

Predictor	Maturation rate (β, P)	2PN rate (β, P)	D3 good embryo rate (β, P)	Blastocyst rate (β, P)	Top-quality blastocyst rate (β, P)
Donor age (years)	-0.04 (0.803)	0.27 (0.204)	0.18 (0.498)	-0.21 (0.446)	0.10 (0.701)
Donor AMH (ng/mL)	-0.59 (0.007)*	0.02 (0.958)	-0.85 (0.023)*	-0.67 (0.092)	-0.08 (0.816)
Donor BMI (kg/m²)	0.33 (0.102)	-0.68 (0.014)*	0.32 (0.374)	-0.12 (0.748)	-0.39 (0.271)
Total FSH dose (IU)	0.00 (0.376)	-0.00 (0.691)	-0.00 (0.172)	0.00 (0.392)	0.00 (0.432)
Peak E2 (pg/mL)	-0.00 (0.837)	-0.00 (0.078)	0.00 (0.890)	-0.00 (0.397)	0.00 (0.943)
P4 (ng/mL)	-0.15 (0.770)	0.61 (0.372)	0.39 (0.653)	-0.39 (0.670)	-0.38 (0.653)
Protocol: PPOS vs GnRH antagonist	2.24 (0.100)	3.59 (0.053)	-5.66 (0.016)*	2.84 (0.255)	-13.60 (<0.001)*
LH use (yes vs no)	-2.11 (0.225)	3.15 (0.182)	1.76 (0.557)	-1.90 (0.552)	-0.50 (0.864)
Dual trigger (vs GnRH-a)	0.09 (0.936)	1.99 (0.206)	-4.43 (0.027)*	-12.19 (<0.001)*	4.99 (0.011)*

β = unstandardized coefficient; values indicate percentage-point change in the outcome per unit of predictor. AMH (per 1 ng/mL); BMI (per 1 kg/m²); total gonadotropin (per 100 IU); protocol (PPOS vs antagonist); trigger (dual vs GnRHa). 95% CI reported for all β.

Primary embryo-quality endpoints use Bonferroni correction (P < 0.005 = significant); 0.005 ≤ P < 0.05 are exploratory. AMH, anti-Müllerian hormone; BMI, body mass index; FSH, follicle-stimulating hormone; LH, luteinizing hormone; E2, estradiol; P4, progesterone; GnRH antagonist, gonadotropin-releasing hormone antagonist; PPOS, progestin-primed ovarian stimulation. *P<0.005 (Bonferroni-adjusted) indicates statistical significance for the five prespecified embryo-quality endpoints; 0.005≤P<0.05 is nominal/exploratory.

Donor AMH was negatively associated with oocyte maturation (β = –0.59; P = 0.007), suggesting that higher ovarian reserve may be linked to reduced maturation efficiency. Donor BMI was the only significant predictor of 2PN fertilization rate, showing a negative association (β = –0.68; P = 0.014), indicating that even in healthy donors, elevated BMI may impair fertilization potential.

Regarding early embryo development, the PPOS protocol was associated with significantly lower Day 3 good-quality embryo rates compared to the GnRH antagonist protocol (β = –5.66; P = 0.016). The use of a dual trigger (GnRH agonist plus low-dose hCG) was also associated with reduced Day 3 embryo quality (β = –4.43; P = 0.027), possibly due to endocrine alterations during oocyte maturation. In terms of blastocyst formation, the trigger method had the most notable impact. Dual trigger was associated with a significantly lower blastocyst formation rate (β = –12.19; P < 0.001), while no other covariates reached significance. Interestingly, for top-quality blastocyst rate, PPOS was again associated with a markedly lower rate (β = –13.60; P < 0.001), whereas dual trigger use was positively associated (β = +4.99; P = 0.011), suggesting that although blastocyst formation rate may be reduced, embryos that develop may be of higher morphological quality.

In summary, AMH, BMI, stimulation protocol, and trigger method were independently associated with embryo quality parameters. Notably, PPOS was consistently linked to inferior embryo morphology, while dual trigger exerted divergent effects depending on the developmental stage assessed.

### 3.4 Propensity score–matched analyses of stimulation protocols and LH supplementation

Propensity score–matched comparisons are summarized in **Table 4** between stimulation protocols (GnRH antagonist vs PPOS) and LH supplementation groups (LH+ vs LH–) are presented in **Table 4A** and **Table 4B**. Effect sizes are reported as absolute differences (percentage points) with 95% confidence intervals, alongside group summaries and P values. Statistical significance for the five prespecified embryo-quality endpoints is defined by Bonferroni (P < 0.005); results with 0.005 ≤ P < 0.05 are exploratory.

**Table 4:** Propensity score–matched comparison of embryo quality outcomes by protocol and LH supplementation.

GnRH antagonist vs PPOS (Matched 154 vs 308 cycles)				
Outcome	GnRH antagonist (n=154)	PPOS (n=308)	Absolute difference, 95% CI	P value
Maturation rate (%)	76.9 ± 13.2	78.1 ± 13.8	1.2 (-1.4 to 3.8)	0.24
2PN fertilization rate (%)	69.8 ± 17.6	74.0 ± 19.0	4.2 (0.7 to 7.7)	0.005
Day 3 good-quality embryo rate (%)	72.8 ± 22.9	67.9 ± 22.7	-4.9 (-9.3 to -0.5)	0.008
Blastocyst rate (%)	60.9 ± 28.5	64.0 ± 24.0	3.1 (-2.1 to 8.3)	0.14
Top-quality blastocyst rate (%)	74.1 ± 21.8	59.7 ± 22.3	-14.4 (-18.6 to -10.2)	<0.001
LH (–) vs LH (+) Supplementation (Matched 81 vs 162 cycles)				
Outcome	LH– (n=81)	LH+ (n=162)	Absolute difference, 95% CI	P value
Maturation rate (%)	78.9 ± 12.9	78.2 ± 14.5	-0.7 (-4.3 to 2.9)	0.66
2PN fertilization rate (%)	69.5 ± 18.2	74.4 ± 19.0	4.9 (-0.0 to 9.8)	0.017
Day 3 good-quality embryo rate (%)	70.4 ± 22.5	69.3 ± 22.1	-1.1 (-7.1 to 4.9)	0.66
Blastocyst rate (%)	63.5 ± 23.5	63.4 ± 25.8	-0.1 (-6.6 to 6.4)	0.96
Top-quality blastocyst rate (%)	69.0 ± 24.7	67.1 ± 20.0	-1.9 (-8.1 to 4.3)	0.45

Comparison of embryo quality outcomes between matched GnRH antagonist and PPOS cycles using 1:2 propensity score matching. Values are presented as mean ± standard deviation. Absolute difference (PPOS − antagonist) is reported in percentage points with 95% confidence intervals. Primary embryo-quality endpoints use Bonferroni correction (P < 0.005 = significant); 0.005 ≤ P < 0.05 are exploratory. 2PN, two-pronuclear zygote; PPOS, progestin-primed ovarian stimulation; GnRH antagonist, gonadotropin-releasing hormone antagonist. Comparison of embryo quality outcomes between matched LH– and LH+ stimulation cycles using 1:2 propensity score matching. Values are presented as mean ± standard deviation. Absolute difference (LH+ − LH−) is reported in percentage points with 95% confidence intervals. Primary embryo-quality endpoints use Bonferroni correction (P < 0.005 = significant); 0.005 ≤ P < 0.05 are exploratory. LH, luteinizing hormone; 2PN, two-pronuclear zygote.

In the protocol-matched cohort (**Table 4A**), 154 GnRH antagonist cycles were matched with 308 PPOS cycles based on donor age, AMH, and BMI. Fertilization rates were significantly higher in the PPOS group (74.0% vs 69.8%; P = 0.005), suggesting enhanced fertilization efficiency. However, this was offset by significantly reduced embryo quality: Day 3 good-quality embryo rate (67.9% vs 72.8%; P = 0.008) and top-quality blastocyst rate (59.7% vs 74.1%; P < 0.001) were both significantly lower in the PPOS group. No significant differences were found in maturation rate (78.1% vs 76.9%; P = 0.24) or overall blastocyst formation (64.0% vs 60.9%; P = 0.14). These findings suggest that although PPOS yields comparable numbers of embryos, their morphological quality may be compromised.

In the LH supplementation cohort (**Table 4B**), 81 FSH-only cycles were matched with 162 cycles receiving added LH, again balanced for donor age, AMH, and BMI. LH supplementation was associated with a significantly higher fertilization rate (74.4% vs 69.5%; P = 0.017), suggesting improved oocyte competence at fertilization. However, no statistically significant differences were found in oocyte maturation (78.2% vs 78.9%), Day 3 good-quality embryo rate (69.3% vs 70.4%), blastocyst formation (63.4% vs 63.5%), or top-quality blastocyst rate (67.1% vs 69.0%) between groups (all P > 0.4).

Together, these findings indicate that while PPOS and GnRH antagonist protocols yield similar oocyte yield and blastocyst formation rates, PPOS is associated with lower embryo quality. Conversely, LH supplementation may modestly enhance fertilization potential without significantly affecting downstream embryo development.

## 4 Discussion

This retrospective analysis of 584 oocyte donor IVF cycles provides meaningful insights into how donor characteristics and stimulation protocols influence oocyte yield and embryo quality. Our findings confirm established predictors—particularly AMH for oocyte quantity—while also uncovering more nuanced associations between stimulation strategies and embryologic outcomes. These results hold practical value for optimizing donor stimulation, especially in balancing treatment efficiency with embryo developmental competence.

### 4.1 Donor AMH and oocyte quantity vs. quality

Anti-Müllerian hormone (AMH) remains the most reliable predictor of ovarian response. In our cohort, each 1 ng/mL increase in AMH was associated with an average increase of approximately one additional retrieved oocyte, consistent with large-scale studies linking AMH to follicular recruitment and response to stimulation (2, 18, 19). As expected, donors with high AMH levels—commonly selected in donor programs—enabled retrieval of numerous oocytes suitable for banking or multiple transfers. However, our results also revealed a modest inverse relationship between AMH and oocyte/embryo quality. Elevated AMH was significantly associated with reduced oocyte maturation and fewer good-quality embryos at both the cleavage and blastocyst stages (20, 21).

### 4.2 Donor BMI and fertilization rate

Interestingly, while donor BMI did not impact oocyte yield or blastocyst quality, it emerged as an independent predictor of impaired fertilization rate. This suggests that increased adiposity may impair oocyte competence, even in otherwise healthy young donors. Previous reports (6, 22) have linked higher donor BMI to decreased pregnancy and live birth rates. Our findings specifically implicate fertilization as the vulnerable stage, possibly due to metabolic dysregulation such as insulin resistance, altered lipid metabolism, or systemic inflammation. Notably, once fertilized, embryos from higher-BMI donors performed comparably in terms of blastocyst formation and morphological quality. This implies that the principal barrier may lie at or before fertilization. These findings underscore the importance of metabolic health prior to donation, even among donors with otherwise normal ovarian function. While many programs already impose BMI cutoffs for donor eligibility, our results suggest that optimizing BMI within the normal range could further improve fertilization outcomes and downstream embryo yield.

### 4.3 Stimulation protocol: PPOS vs. GnRH antagonist

Although PPOS offers logistical advantages and has been associated with comparable pregnancy outcomes in some studies (8), our data suggest potential embryologic disadvantages in young donor populations. The choice of ovarian suppression protocol had no significant effect on oocyte yield but had a notable impact on embryo quality. Compared to GnRH antagonist cycles, PPOS cycles resulted in 6% fewer Day 3 good-quality embryos and 13% fewer top-quality blastocysts, as shown by both multivariable and propensity-matched analyses. These findings align with previous reports (9, 23).

The likely mechanism involves supraphysiologic progesterone exposure impairing oocyte competence through altered granulosa cell signaling (24), disrupted oocyte gene expression and mitochondrial function (25), or impaired cumulus–oocyte communication (26). Additionally, more complete LH suppression in PPOS may disturb steroidogenesis compared to the partial suppression in antagonist cycles (27).

Although one smaller donor study reported similar oocyte and embryo counts between protocols (28), embryo quality was not evaluated. In our larger cohort, quality differences were evident despite comparable oocyte and blastocyst yields. Since all embryos were frozen before transfer, our findings reflect intrinsic differences in oocyte and embryo quality rather than endometrial receptivity.

Clinically, PPOS provides logistical and cost advantages, especially in donor settings where scheduling flexibility and avoiding daily injections are desirable (29). However, when maximizing embryo competence is the priority—for instance, in egg banking or efforts to maximize cumulative live birth per retrieval—GnRH antagonist protocols may be preferable. Our data suggest caution in assuming equivalence between protocols, and further studies should evaluate whether modifications to PPOS (e.g., progestin type, dose, or trigger timing) could mitigate its potential impact on embryo quality (30–32).

In our clinical workflow, the first transfer is intentionally performed with the top-quality embryo, which reduces the likelihood of detecting donor protocol/trigger effects at the first-transfer level. For translational relevance, cumulative live birth per retrieval may be a more sensitive endpoint if one protocol yields more transferable embryos. We plan to analyze cumulative outcomes once longitudinal follow-up is complete.

### 4.4 LH supplementation: limited utility in young donors

In our cohort, routine LH supplementation did not enhance oocyte maturation or embryo development. Although LH+ cycles showed slightly higher fertilization rates, the difference was not statistically significant after adjustment. This supports previous findings suggesting that LH supplementation may benefit selected populations—such as older women or those with hypogonadotropic hyporesponsiveness (12, 33, 34)—but not necessarily young, normo-ovulatory donors. In such donors, endogenous LH levels are typically sufficient even under GnRH antagonist protocols, rendering exogenous LH unnecessary (35).

Moreover, excessive LH exposure may negatively affect follicular synchrony or promote premature luteinization (36). Consistent with this, we observed a modest inverse association between total LH dose and oocyte yield. Overall, FSH-only stimulation appears adequate for most young donors. LH supplementation may be selectively considered in cases of prior suboptimal fertilization or borderline ovarian reserve, but its routine use may not be necessary in young normo-responders. This approach may reduce medication costs and simplify protocols without compromising clinical outcomes.

### 4.5 Trigger strategy: GnRH agonist alone vs. dual trigger

Dual trigger—combining a GnRH agonist with low-dose hCG—was initially designed to improve oocyte maturation, particularly in poor responders. However, in our high-responder donor cohort, dual trigger was not associated with better oocyte or embryo outcomes (37). In fact, it was linked to significantly lower blastocyst formation rates and slightly reduced Day 3 embryo quality. Although the proportion of top-quality blastocysts was marginally higher, the absolute number of blastocysts was lower, suggesting a trade-off between embryo quality and quantity.

One possible explanation is selection bias, as dual trigger was often used in donors with fewer mature follicles or prior poor maturation. However, our adjusted models and matched analyses support a true physiological effect. Biologically, hCG prolongs LH receptor activation in granulosa cells, which may promote early luteinization or disrupt follicular signaling in ways that impair oocyte competence.

Given that GnRH agonist trigger alone achieved high maturation and embryo development rates while effectively reducing OHSS risk, it remains the preferred strategy for most donor cycles. Dual trigger may still be appropriate for specific indications such as prior poor oocyte maturation or asynchronous follicular development, but it does not confer consistent benefit in high-responder donors (38, 39).

### 4.6 Strengths and limitations

Key strengths of this study include its large donor cohort, use of standardized laboratory protocols, and robust statistical methodology, including multivariable adjustment and propensity score matching. The exclusive focus on donor cycles ensured a homogenous population, minimizing uterine-related confounding and enabling clearer evaluation of oocyte and embryo-level outcomes.

This study was conducted at a single high-volume IVF center, which ensured standardized clinical management and laboratory workflows but may limit the external generalizability of our findings to other populations or practice environments. Future multi-center studies including diverse donor and recipient populations are warranted to validate these associations. Additionally, the retrospective design precludes causal inference and introduces potential selection bias, particularly regarding stimulation protocol selection. Clinical decisions may have been influenced by physician preference or logistical constraints. We also did not stratify outcomes by extreme AMH levels, which could differentially affect embryo quality. While statistically significant, the absolute decrease in fertilization rate with increasing BMI was modest, and the clinical relevance of this finding warrants cautious interpretation. As our embryo assessment relied on morphological criteria rather than genetic analysis in this analysis, the relationship between stimulation protocols and euploidy remains uncertain. Further studies integrating PGT-A data are needed to determine whether the observed morphological differences reflect true differences in embryo euploidy. Furthermore, recipient outcomes were reported in aggregate and not stratified by stimulation protocol, due to heterogeneity in recipient characteristics and embryo origin. Future prospective or randomized studies—particularly those evaluating blastocyst yield, embryo aneuploidy, and recipient outcomes—are warranted to validate and expand upon these findings. Another limitation is that calendar time was not modeled explicitly; although clinical and embryology workflows were highly standardized and endpoints were laboratory-based, any residual secular/batch effects cannot be excluded. Because baseline data for cycles excluded at screening were not captured in the research registry, we could not compare included versus excluded cohorts. Although exclusions were procedural and occurred prior to outcome ascertainment, some selection bias cannot be excluded. Future prospective, multi-center registries that capture all screened donors are needed to directly evaluate this issue. To preserve common support and avoid overfitting in a finite matched sample, the propensity-score model was intentionally limited to baseline determinants (age, AMH, BMI) measured prior to stimulation; trigger-day estradiol and total gonadotropin (on-path variables), non-uniform follicle counts, and calendar year were not included in the PS but were handled in multivariable outcome models and acknowledged in the Limitations. Our matched cohort sizes and observed variance provide approximately 80–85% power to detect approximately 6% absolute differences in embryo-quality rates; larger effects (e.g., top-quality blastocyst rate) are estimated with good precision, while approximately 5% effects are near the detection boundary. Interval estimates and Bonferroni-adjusted inferences are provided to contextualize statistical uncertainty.

## 5 Conclusion

This study confirms that donor AMH is the principal predictor of oocyte yield, while embryo quality is influenced by both donor characteristics and stimulation protocols. Higher donor AMH levels and elevated BMI were modestly associated with reduced oocyte maturation and fertilization rates, underscoring the importance of tailoring stimulation strategies to individual donor profiles. Although PPOS yielded comparable oocyte yield to the GnRH antagonist protocol, it was consistently associated with lower embryo quality, warranting caution when embryo competence is a clinical priority. LH supplementation demonstrated limited benefit, and dual trigger did not improve outcomes compared to GnRH agonist alone in this young donor cohort. All protocols achieved high pregnancy and live birth rates, reflecting the overall efficacy of donor IVF. Nevertheless, individualizing stimulation strategies based on AMH, BMI, and prior cycle response may enhance oocyte yield and embryo developmental potential. Further prospective studies are warranted to validate these associations and inform optimal protocol selection in clinical donor IVF programs.

## Data Availability

The original contributions presented in the study are included in the article/supplementary material. Further inquiries can be directed to the corresponding author.
